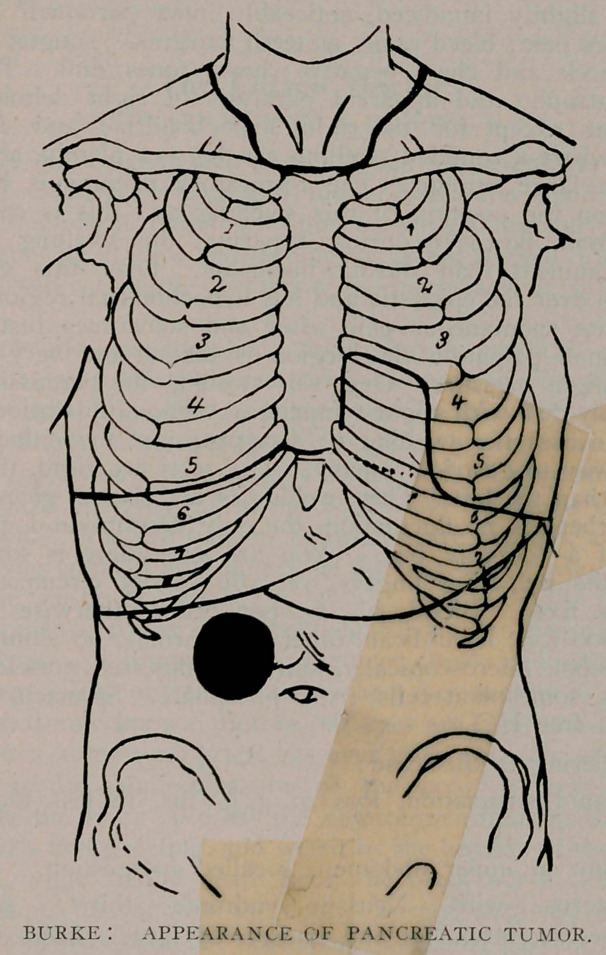# Diagnosis of Pancreatic Affections1Read at the meeting of the Medical Society of the Counfy of Erie February 15, 1909.

**Published:** 1909-04

**Authors:** Joseph Burke

**Affiliations:** Visiting Surgeon, Emergency Hospital, Buffalo, N, Y.


### Diagnosis of Pancreatic Affections[1]

By JOSEPH BURKE, D. Sc., M. D.
Visiting Surgeon, Emergency Hospital, Buffalo, N. Y.

THE right upper quadrant of the abdomen has at all times defied the efforts of the diagnostician to dogmatically differentiate the diseases of the organs contained within its limits, mostly because these organs in one way or another have anatomical and physiological relations in common. Thus the pylorus, the duodenum, the gall bladder and bile ducts and the pancreas, are topographically akin and in many cases in disease of any one of these organs, the symptoms as pain, icterus, local tenderness, and the like, that arise from it, cannot always be definitely interpreted as arising from that special organ alone, but can

1. Read at the meeting of the Medical Society of the Counfy of Erie February 15, 1909.

just as well be attributed to all, thus it is that the diagnosis of disease of the pancreas, the most concealed organ of all, is not on a firm clinical footing. The report, therefore, of any case of pancreas disease needs no apology for its addition to an already too scant literature.

Up to the time when von Mering and Minkowski published the results of their experiments upon animals, in which they partially and totally extirpated the pancreas, very little was known as to the symptoms of disease of this organ and hence little attention was given to the diagnosis; but when they established the fact that any functional derangement or impairment of the pancreas—namely, a diminution or absence of the pancreatic secretion, would give rise to glycosuria and rapid progressive emaciation, as well as a diminution or absence of indican in the urine, they undoubtedly laid the foundation of our present knowledge of the symptoms that result in an individual whose pancreas is the seat of disease. Add to these three, a fourth, icterus, and you have the classical quartet of symptoms that usually accompany affections of the pancreas. Lately there has come a urinary reaction—I believe it has come to stay—the so-called Cammidge test, especially the improved "C" reaction, a test though at present in an experimental stage, bids fair to become pathognomonic in the chronic inflammatory troubles. As a sixth symptom is to be mentioned the altered condition of the stools which, as a rule, are copious and contain starch granules undigested muscle fibers, and quantities of fat globules.

Concerning icterus in pancreas disease I am going to state that any one who can diagnosticate the cause of icterus in every given case, is certainly master of the differential diagnosis of diseases of the organs in the upper abdomen generally, but such a master has not yet appeared in our science or generation. However, I shall tax your patience with the following clinical "icteric" syndrome of Neusser, that is, in any case of icterus where the following symptoms are also present, (a) thirst, (b) large appetite, (c) copious stools which contain starch, undigested muscle fibers, and fat, (d) no bradycardia, (e) no indican in the urine, and (f) polyuria without sugar—other things being equal, our attention is directed to the extreme probability that a disease of the pancreas confronts us. This syndrome is remarkably shown in a case that recently came under my observation in which on the basis of it, with other findings, I was able to make a correct diagnosis of an affection of the pancreas, which case I shall report here tonight. There are two other symptoms—namely, sialorrhea and persistent vomiting, that are present in some cases; obstinate constipation is said to characterise acute hemorrhagic pancreatitis. I do not know of any better way to discuss the local physical manifestations of pancreas affections than by the report of a personal case, which follows:

Mrs. Y., married, never pregnant; had pertussis and measles when 5 years of age; diphtheria at 14; rheumatism at 19; family history negative. For the past few years has complained at

BURKE: APPEARANCE OF PANCREATIC TUMOR.

various times of attacks of indigestion. September, 1908, suddenly in the night, suffered severe pain in epigastrium, fainting spells, and the like, which were ascribed to acute indigestion. Since then has been troubled more or less with stomach and pains in the right shoulder.

At present she complains of pain in epigastrium immediately after and sometimes just before eating. Does not vomit. Complains of severe pain in right shoulder, is unable on account of pain to raise arm. Appetite good, hunger present nearly always, but on account of pain is afraid to eat. Complains of thirst, must arise several times during the night to drink and to urinate; bowels move freely, stools copious and light colored. In September, 1908, her weight was 158 lbs., is now 126½ (in less than five months a loss of 31½ lbs).

*Physical Examination.*—Pulse, 78; respiration, 20; temperature, 98½° F. Patient is pale, sallow; skin lax, fat poor. Conjunctivæ slightly jaundiced, noticeably inner portions. Mucous membranes pale; bleed easily at teeth margins. Tongue slightly coated; neck and chest negative; heart tones dull. There is marked atrophy and apparent paralysis of right deltoid. Abdomen flat except for the right hypochondriac and epigastic regions, where a rounded swelling can be seen plainly, about the size of a large orange. The respiratory excursions have no effect upon the position of this swelling and this is confirmed by palpation, however during experium the swelling appears more prominent than during inspirium. Over this swelling, as well as over the epigastic and left hypochondrial regions there is exquisite spontaneous pain after and sometimes just before eating; upon palpation this region is tender and very painful to even slight pressure. Over this swelling the percussion note is distinctly dull with slight tympany. Artificial dilatation of the stomach causes the swelling to disappear and the dulness over it gives way to gastric tympany. The recti are rigid, the right more so than the left. This swelling is not elastic, gives no resistance, there is no fluctuation, the only description I can give is that of a "doughy feel." You are sure there is something beneath the palpating fingers, yet you cannot circumscribe it. Tumor is fixed. Blood, hb. 65 per cent., otherwise normal. Urine, 2600 c.c.; no indican, otherwise normal; no albumin; no sugar. Stool, microscopically, starch, undigested muscle fibers, fatty cells, some plant cells, triple phosphate. Stomach, delayed digestion; free HCl.

Considering in this case:

1. Rapid emaciation, loss of 31½ lbs. in less than four months.

2. Pain in upper abdomen, socalled indigestion.

3. Icterus, with Neusser syndrome: thirst, polyuria, large appetite, starch, fat and undigested muscle fibers in copious stools, pulse, 78, and absence of indican in the urine.

4. Local manifestations, fixed swelling in right hypochondriac and epigastric regions, whose dulness upon percussion gave way to gastric tympany after artificial dilatation of the stomach, and disappearance of the swelling at the same time you have a classical clinical picture of an affection of the pancreas, which could be mistaken for nothing else, a positive diagnosis which was confirmed by the findings at operation. I found after opening the gastro-hepatic omentum that the pancreas head was swollen about the size of an orange, grayish pink in color; the remainder of the organ was also swollen. The gallbladder was inflamed but normal in size and contained a very black, thick muco-purulent bile, so thick that it could not run through canula of aspirator. I found no stones in the common duct. Cholecystotomy completed the operation.

1092 MAIN STREET.

## Figures and Tables

**Figure f1:**